# Optimization of protoplast cell division and microcallus formation toward somatic embryogenesis in oil palm

**DOI:** 10.3389/fpls.2026.1952622

**Published:** 2026-10-07

**Authors:** Mohamed A. A. Ahmed, Hanlu Su, Elboghdady A. E. A., Seerat Fatima, Zhiying Li, Yong Wang

**Affiliations:** 1 State Key Laboratory of Tropical Crop Breeding/Coconut Research Institute/Sanya Research Institute, Chinese Academy of Tropical Agricultural Sciences, Sanya, Hainan, China; 2 Plant Production Department (Horticulture - Medicinal and Aromatic Plants), Faculty of Agriculture (Saba Basha), Alexandria University, Alexandria, Egypt; 3 College of Horticulture and Forestry Sciences, Huazhong Agricultural University, Wuhan, Hubei, China; 4 Tropical Fruits Research Department, Horticulture Research Institute, Agricultural Research Center, Giza, Egypt

**Keywords:** cell division, microcallus, microcolony, protoplast, somatic embryogenesis

## Abstract

**Background:**

Efficient protoplast regeneration remains a major obstacle to the application of somatic hybridization, genome editing and precision breeding in oil palm (*Elaeis guineensis*); while early developmental stages, from cell wall regeneration and cell division to microcolony and microcallus formation, could be more important, which could be connected easily with the traditional tissue culture system for succeeding plant regeneration. This study aimed to optimize the protopalst culture conditions for cell division and microcallus formation by identifying the most effective combinations of culture systems, basal media and plant growth regulators.

**Methods:**

Protoplasts isolated from different oil palm tissues (embryogenic callus, suspension cultures, and somatic embryos) were investigated by protoplast yield and viability; protoplasts from the embryogenic callus showed the most satisfactory response and was therefore used for subsequent optimization of culture conditions. The protoplasts were cultured using six culture systems (liquid, droplet, agarose pool, well-plate culture, feeder layer, and nurse culture) in combination with eight basal media (BH3, MS, WPM, Y3, KM, B5, N6, and KPR), and the culture effects were evaluated based on protoplast viability, plating efficiency, cell division, microcolony and microcallus formation. Microcallus emerged from the KPR solid medium were transferred to previously established traditional tissue culture system for further somatic embryogenesis and regeneration.

**Results:**

BH3 liquid medium was particularly effective for early protoplast recovery and rapid cell wall regeneration, whereas KPR solid medium combined with the nurse culture system, provided the most favorable conditions for sustained cell division, plating efficiency, microcolony and microcallus formation. Cell division occurred within 3–7 days, followed by microcolony formation after approximately 7 days and visible microcallus formation after 60 days. Microcallus were transferred for further development, somatic embryos and bud regeneration was observed after 90 days and 120 days, respectively.

**Conclusions:**

The present study focused on the early developmental stages of oil palm protoplast culture, and optimized a stage-specific culture strategy for quick microcallus formation; combined with previous oil palm tissue culture system, further somatic embryogenesis and bud regeneration were established successfully. This work not only promoted the protoplast regeneration of oil palm, but also provides a promising strategy for other tropical palm trees such as coconut and betel nut.

## 1 Introduction

Oil palm (*Elaeis guineensis* Jacq.) is among the most economically important perennial crops worldwide and is the highest-yielding oil-producing species among cultivated oil crops. Its exceptional productivity supports global food supply chains and contributes substantially to industrial processing, oleochemical manufacturing, and renewable biofuel production. However, sustainable production is increasingly threatened by environmental pressures, emerging diseases, and climate-related abiotic stresses. Genetic improvement through conventional breeding remains constrained by long breeding cycles, extensive heterozygosity, and a prolonged juvenile phase that may exceed 10 years before reliable trait evaluation becomes possible (Alhaji et al., 2024; Manorama et al., 2024; Ali et al., 2024; Rahman et al., 2025; Purnama et al., 2025).

Recent advances in plant biotechnology have highlighted protoplast-based technologies as versatile platforms for crop improvement. Protoplasts, plant cells lacking cell walls, offer unique opportunities for somatic hybridization, transient gene expression, cellular reprogramming, and genome editing. Their compatibility with the direct delivery of nucleic acids and ribonucleoprotein complexes makes protoplast systems particularly attractive for potential future applications of CRISPR/Cas-based genome editing. In addition, protoplast fusion offers an effective approach to overcoming sexual incompatibility barriers and facilitating the incorporation of desirable agronomic traits (Yue et al., 2021). However, successful protoplast isolation and regeneration are strongly influenced by multiple biological and technical factors, including species, genotype, tissue source, developmental stage, enzymatic composition, osmotic conditions, isolation procedures, and incubation conditions (Davey et al., 2005).

Despite their considerable potential, efficient regeneration from isolated protoplasts remains a major barrier to the broader implementation of protoplast-assisted breeding technologies. This challenge is particularly pronounced in woody monocotyledonous species such as tropical palms, where slow cellular reprogramming, sensitivity to oxidative stress, restricted embryogenic competence, and limited post-isolation cell division substantially reduce regeneration efficiency (Wallin and Johansson, 1990; Umate et al., 2005; Azad et al., 2006).

The six culture configurations were selected to represent complementary physical and biological strategies for supporting isolated protoplasts during the transition from single cells to multicellular colonies. Liquid culture was included as a conventional unrestricted culture environment, whereas droplet culture was selected to provide localized cell concentration while maintaining a liquid environment. Agarose-pool culture was included to evaluate the effect of semi-solid immobilization on cell stability and cell–cell proximity. Well-plate culture was evaluated as a spatially confined system designed to reduce cell dispersion and maintain a localized nutrient environment. Feeder-layer culture was selected to assess the potential contribution of supporting cells through a conditioned cellular environment, whereas nurse culture was included as a distinct cell-supported system intended to provide diffusible nutritional and signaling factors during early protoplast development. Thus, the six systems were not selected arbitrarily; they were deliberately chosen to compare progressively different physical constraints and degrees of cellular support. This experimental design enabled us to determine whether early oil palm protoplast development was primarily favored by unrestricted liquid exposure, spatial confinement, semi-solid immobilization, or a conditioned cellular environment.

In oil palm, previous studies have reported advances in transient transformation and PEG-mediated delivery systems; however, reproducible regeneration protocols capable of sustaining early cell division and microcallus formation remain limited. Earlier regeneration attempts using embryogenic suspension cultures and agarose-based culture systems achieved only partial success and often exhibited variable responses across experiments (Masani et al., 2013). Similar challenges have also been reported in other tropical palm species, including areca nut, despite recent advances in genomic resources and transformation-related research (Wei and Wei, 2024). Previous studies have demonstrated that protoplast development is influenced by several factors, including tissue origin, genotype, enzymatic digestion conditions, osmotic regulation, plating density, basal medium composition, and culture configuration. Among these factors, feeder-layer and nurse culture systems have shown potential to enhance cell division and microcolony establishment by providing a conditioned growth environment and facilitating intercellular signalling; however, their effectiveness in tropical palm species remains insufficiently investigated (Masani et al., 2013).

Previous studies have established the feasibility of oil palm protoplast culture and, importantly, have demonstrated that complete plant regeneration from oil palm protoplasts is possible under optimized conditions (Ravigadevi et al., 1996; Masani et al., 2013). These studies represent important milestones and provide the foundation for subsequent applications of oil palm protoplast technology. However, the available protocols have generally focused on achieving regeneration under specific optimized conditions rather than systematically dissecting the contribution of different culture configurations to the successive early developmental stages of isolated protoplasts. A critical unresolved issue is therefore not whether oil palm protoplasts can regenerate, but how the physical culture environment and basal medium interact to determine the probability and timing of successive developmental transitions following isolation. In particular, comparative information remains limited regarding the relative performance of liquid, droplet, agarose-pool, well-plate, feeder-layer, and nurse-culture systems when evaluated using the same experimental framework and developmental endpoints.

The present study addresses this gap by systematically comparing six distinct culture configurations together with multiple basal media and by following protoplast development sequentially from cell-wall regeneration through first and second cell division, plating efficiency, microcolony establishment, and microcallus formation. The novelty therefore lies not in claiming the first oil palm microcallus formation or the first complete regeneration of oil palm plants from protoplasts, but in establishing a stage-specific comparative framework that identifies which combinations of medium and culture configuration are most supportive at successive stages of early protoplast development. This distinction is particularly important because the present results demonstrate that no single culture condition was uniformly optimal across all developmental endpoints.

Although previous studies have reported partial success in oil palm protoplast regeneration, reproducible early-stage regeneration remains limited, particularly in optimizing culture media and conditions to support sustained cell division and microcallus development. In the present study, different basal media and protoplast culture systems were systematically evaluated to identify conditions favorable for early-stage protoplast development in oil palm. Initial screening experiments were performed using multiple culture approaches, followed by a detailed assessment of the most responsive systems. Particular emphasis was placed on improving protoplast viability, promoting cell wall regeneration and early cell division, enhancing plating efficiency, and inducing microcolony and microcallus formation. The present study establishes a stage-specific framework for improving early oil palm protoplast development up to microcallus formation. Although these optimized conditions may provide a useful starting point for subsequent protoplast-based applications, the present study did not evaluate genetic transformation, CRISPR-mediated genome editing, somatic hybridization, or regeneration of whole plants. These applications therefore remain important directions for future investigation. Future work should determine whether the optimized early-stage conditions can be integrated with transformation or genome-editing procedures and subsequently extended toward embryogenic development, shoot formation, and whole-plant regeneration.

## 2 Materials and methods

### 2.1 Plant materials

Oil palm (*Elaeis guineensis* Jacq.) embryogenic callus, cell suspension cultures, and somatic embryo-derived tissues were used as explant materials for protoplast isolation. All plant materials were obtained from the Coconut Research Institute of the Chinese Academy of Tropical Agricultural Sciences (CRI-CATAS), China. The embryogenic callus originated from early-stage floral buds collected before opening of the inflorescences and was maintained as a single callus line. Friable embryogenic callus was used 7 days after subculture/renewal, with approximately 1 g of callus used for each protoplast isolation. Prior to protoplast isolation experiments, the callus was subcultured approximately 2–3 times, as required to maintain actively growing and viable tissue. The available records identified the plant material only as *Elaeis guineensis* Jacq.; no specific genotype or accession information was available. Healthy and actively growing tissues were selected to ensure high protoplast yield and viability.

### 2.2 Isolation and purification of protoplasts

Protoplast isolation was adapted from previously published protocols with minor modifications to improve recovery and viability in tropical palm tissues (Guo et al., 2023; Yoo et al., 2007).

Fresh materials were excised into 0.5–1.0 mm segments under sterile conditions. Approximately 1 g tissue was incubated in 20 mL enzyme solution containing 0.3% Cellulase Onozuka R-10, 0.15% Macerozyme R-10, and 0.20% pectinase prepared in W5 buffer supplemented with BSA, AMP, and β-mercaptoethanol (**Supplementary Table S1**). Enzyme mixtures were pre-incubated at 55 °C for 10 min.

Digestion was performed in darkness at 28 °C under agitation (60 rpm) for 5 h. All solutions were sterilized by 0.22 μm membrane filtration (Zhang et al., 2011; Hu et al., 2015).

Following digestion, suspensions were diluted with chilled W5 solution, filtered through 70 μm mesh, and centrifuged at 150 × g for 5 min at 4 °C with as described by Yoo et al. (2007). Pellets were washed three times using chilled W5 and resuspended in 2 mL W5 solution. Purified protoplasts were stabilized on ice for 20 min before downstream analysis and culture. Isolation efficiency and morphology were evaluated using a Zeiss Axio Imager M2 microscope.

### 2.3 Assessment of protoplast yield and viability

Protoplast yield and viability were determined immediately after isolation. Total protoplast yield was determined using a hemocytometer and expressed as the number of protoplasts per gram fresh weight (cells g^-^¹ FW).

The yield of isolated protoplasts (cells g^-^¹ fresh weight) was calculated using the formula:


$$\text{Protoplast yield}=\frac{\text{Total number of protoplasts}}{\text{Fresh weight of tissue used }(\text{g})}$$

Protoplast viability was evaluated using the fluorescein diacetate (FDA) staining assay following the method described by (Henn et al., 1998; Costa et al., 2018), with minor modifications.

A stock solution was prepared by dissolving 5 mg FDA in 1 mL acetone. Subsequently, 100 μL of freshly isolated protoplast suspension was mixed with 1 μL of FDA solution to obtain a final concentration of 0.01%. Samples were gently mixed and incubated for 5 min in darkness at room temperature.

Viable protoplasts exhibiting intact plasma membrane integrity hydrolyzed FDA and emitted green fluorescence under fluorescence microscopy, whereas non-fluorescent cells were considered non-viable.

The percentage of viable protoplasts was calculated as:


$$\text{Viability }(\%)=\frac{\text{Number of viable protoplasts}}{\text{Total number of protoplasts}}\times 100$$

### 2.4 Evaluation of different protoplast culture systems

To determine the most suitable conditions for tropical palm protoplast development and regeneration, different culture systems and basal media were systematically evaluated. The experiments aimed to optimize protoplast survival, cell wall regeneration, cell division, microcolony formation, and microcallus induction.

Freshly isolated protoplasts were washed and allowed to stabilize for 20 min at room temperature prior to culture initiation. Protoplast density at plating was adjusted according to the explant source and was maintained across the tested culture systems: 7.9 × 10^6^ cells mL^-^¹ for embryogenic callus, 1.74 × 10^6^ cells mL^-^¹ for cell suspension cultures, and 5.48 × 10^6^ cells mL^-^¹ for somatic embryo-derived tissues.

Six different protoplast culture systems were comparatively investigated based on previously established protocols with modifications (Kaur et al., 2006; Hu et al., 2015; Dai et al., 2013; Sushamakumari et al., 2000) (**Supplementary Figure S1** and **S2**). All cultures were maintained in darkness at 28 °C under aseptic conditions.

#### 2.4.1 Liquid culture

Purified protoplasts were suspended directly in liquid culture medium and cultured in sterile Petri dishes containing 1 mL of medium. Cultures were monitored regularly for cell wall regeneration, cell division, and microcolony formation.

#### 2.4.2 Droplet culture

For droplet culture, protoplast suspensions were distributed as small liquid droplets on sterile Petri dishes according to Kitamura (1993). The droplets were maintained under controlled humidity throughout the culture period. Unlike agarose bead culture, protoplasts remained suspended in liquid droplets without immobilization within a semi-solid matrix.

#### 2.4.3 Agarose pool culture

For agarose pool culture, protoplasts were embedded in a semi-solid agarose matrix according to Hu et al. (2015). Molten culture medium supplemented with agarose was gently mixed with protoplast suspensions and allowed to solidify under sterile conditions before incubation.

#### 2.4.4 Well-plate culture

A well-plate culture system was developed and evaluated to improve protoplast aggregation, spatial confinement, and exposure to a controlled nutrient environment during the early stages of culture. Purified protoplasts were suspended in culture medium and dispensed into individual wells containing a defined volume of medium, allowing the cells to remain concentrated within a restricted culture area while maintaining continuous contact with the surrounding nutrient solution.

The well-plate configuration facilitated uniform distribution of protoplasts, reduced cell dispersion, and enabled regular microscopic observation of cell wall regeneration and subsequent cell divisions. Cultures were maintained under the same environmental conditions described above and were monitored periodically for protoplast survival, cell division, microcolony formation, and early microcallus development.

#### 2.4.5 Feeder layer culture

Feeder-layer cultures were established using actively growing oil palm suspension cells prepared according to (Raikar et al., 2006). Suspension cells harvested four days after subculture were mixed with double-strength KPR medium containing 0.8% (w/v) SeaPlaque agarose. Approximately 10 mL of feeder medium was dispensed into sterile Petri dishes and conditioned for 24 h prior to inoculation.

A sterile membrane filter (47 mm diameter, 0.2 μm pore size) was placed on the feeder layer surface, and 200 μL of protoplast suspension was evenly distributed onto the membrane. Colony formation frequency was recorded after 40 days of culture.

#### 2.4.6 Nurse culture

For nurse culture, purified protoplasts were embedded in 0.8% (w/v) SeaPlaque agarose supplemented with KPR medium at a density of approximately 1 × 10^5^ cells mL^-^¹. The agarose-protoplast mixture was prepared either as thin layers (5 mL per dish) or as individual droplets (100 μL each, 5–6 droplets per dish). Cultures were maintained under the same environmental conditions described above and evaluated for cell division, plating efficiency, and microcolony formation.

### 2.5 Evaluation of different basal media

To identify culture conditions favorable for early protoplast development, we evaluated eight culture media formulations: BH3, Murashige and Skoog (MS), Woody Plant Medium (WPM), Kao and Michayluk medium (KM) (Kao and Michayluk, 1975), Y3 medium, N6 medium, Gamborg B5 medium, and KPR medium (Gamborg et al., 1968). The evaluated formulations included their corresponding supplements, osmotic regulators, and plant growth regulator combinations, as summarized in **Supplementary Table S2**. Protoplasts were cultured at the specified plating densities using the designated culture systems, and developmental responses were monitored regularly by light microscopy.

The evaluated parameters included cell wall regeneration, first and second cell division, microcolony formation, and microcallus development. The objective of this experiment was to identify culture media formulations capable of supporting sustained mitotic activity and early colony establishment under oil palm protoplast culture conditions.

### 2.6 Determination of plating efficiency

The efficiency of protoplast development was assessed by calculating plating efficiency (PE%), defined as the percentage of plated protoplasts capable of forming microcolonies.

The plating efficiency was calculated as follows:


$$\text{PE (\%)}=\frac{\text{Number of microcolonies formed}}{\text{Total number of protoplasts plated}}\times 100$$

Each treatment consisted of three independent biological replicates, and all experiments were repeated independently to ensure reproducibility.

### 2.7 Microcallus induction and somatic embryogenesis

Following initial cell wall regeneration and mitotic activation, developing protoplast cultures that exhibited sustained cell division were transferred to optimized conditions to support microcolony expansion and microcallus induction. Cultures were maintained under dark conditions at 28 °C and regularly monitored using inverted light microscopy.

Microcolony formation was defined as cell aggregates consisting of 3–7 cells, whereas structures containing eight or more cells were classified as advanced microcolonies. Microcallus formation was defined as compact and organized cell clusters that were visibly detectable under microscopic observation after prolonged culture.

To facilitate the transition from active cell division to sustained cell proliferation, osmotic conditions were gradually reduced by transferring cultures to a modified medium based on BH3 supplemented with 0.15 M osmoticum (EME-based formulation) in a 2:1 ratio with the original BH3 medium. This step was used to promote cell stability and continued division during early microcolony development.

For further development, developing microcalli were transferred onto solid regeneration media consisting of MS, Y3, or N6 basal formulations supplemented with 3% (w/v) sucrose and 0.8% (w/v) agar, together with specific plant growth regulator combinations (**Supplementary Table S3**). Cultures were then incubated under dark conditions at 28 °C until embryogenic callus-like structures were observed. Succeeding microcallus development and somatic embryogenesis were done based on our previous oil palm tissue culture procedures (Zhang et al., 2021).

### 2.8 Microscopy and statistical analysis

Cell viability and regeneration responses were evaluated using light, fluorescence, and confocal microscopy. FDA fluorescence was detected using a Nikon SMZ18. GFP detection was performed using EVOS M5000. Confocal imaging was conducted using Zeiss LSM 900. Quantitative data were expressed as mean ± SD (n = 3), based on three independent biological replicates, with each independent protoplast isolation considered an experimental unit. For experiments involving two experimental factors, such as basal medium and culture system, data were analyzed using two-way factorial ANOVA, including the main effects of basal medium, culture system, and their interaction (basal medium × culture system). Significant differences among means were evaluated using Tukey’s HSD test at p < 0.05. For experiments involving a single experimental factor, one-way ANOVA followed by Tukey’s HSD test was used at p < 0.05.

## 3 Results

### 3.1 Optimization of protoplast isolation

Protoplasts were successfully isolated from embryogenic callus, suspension cultures, and somatic embryo-derived tissues of oil palm using the optimized enzymatic digestion procedure. Freshly isolated protoplasts exhibited intact morphology and remained structurally stable following purification.

Among the materials, embryogenic callus of oil palm released the highest number of protoplasts 7.98 × 10^6^ cells/g FW, the viability of protoplasts was 85.40% for the callus. FDA staining in **Figure 1** confirmed the presence of metabolically active cells immediately after isolation, as indicated by strong green fluorescence signals.

**Figure 1:**
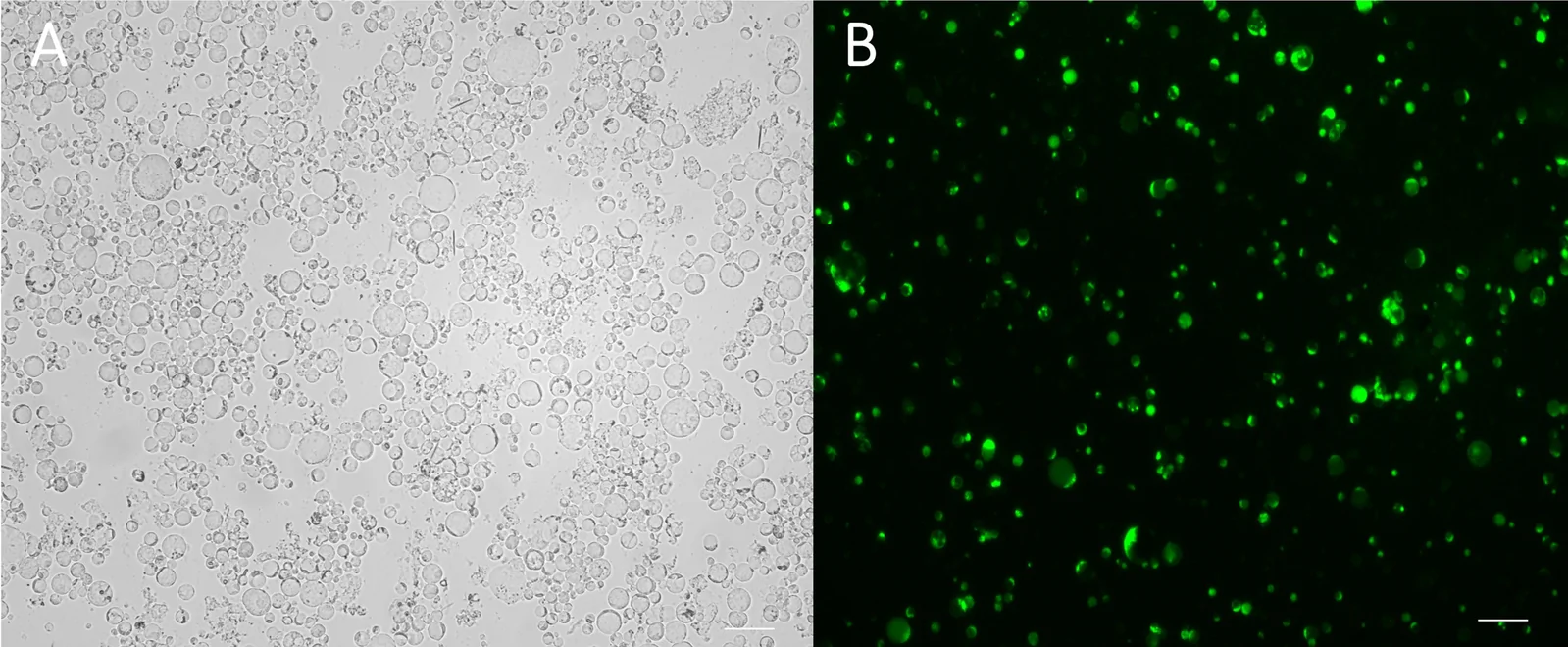
Protoplast isolation from embryogenic callus of oil palm. **(A)** Protoplasts isolated from the callus—white light. **(B)** Protoplasts isolated from the callus and labeled with FDA—UV light. Bar = 50 µm.

### 3.2 Early cell division after culture initiation

Morphological observations demonstrated progressive cellular development during protoplast culture. Cell wall regeneration was initiated within 2–9 days depending on the basal medium, followed by the first mitotic division occurring as early as 3–7 days in BH3 medium. Subsequent second divisions occurred between 6 and 10 days of culture, leading to the formation of small multicellular aggregates (**Figure 2**).

**Figure 2:**
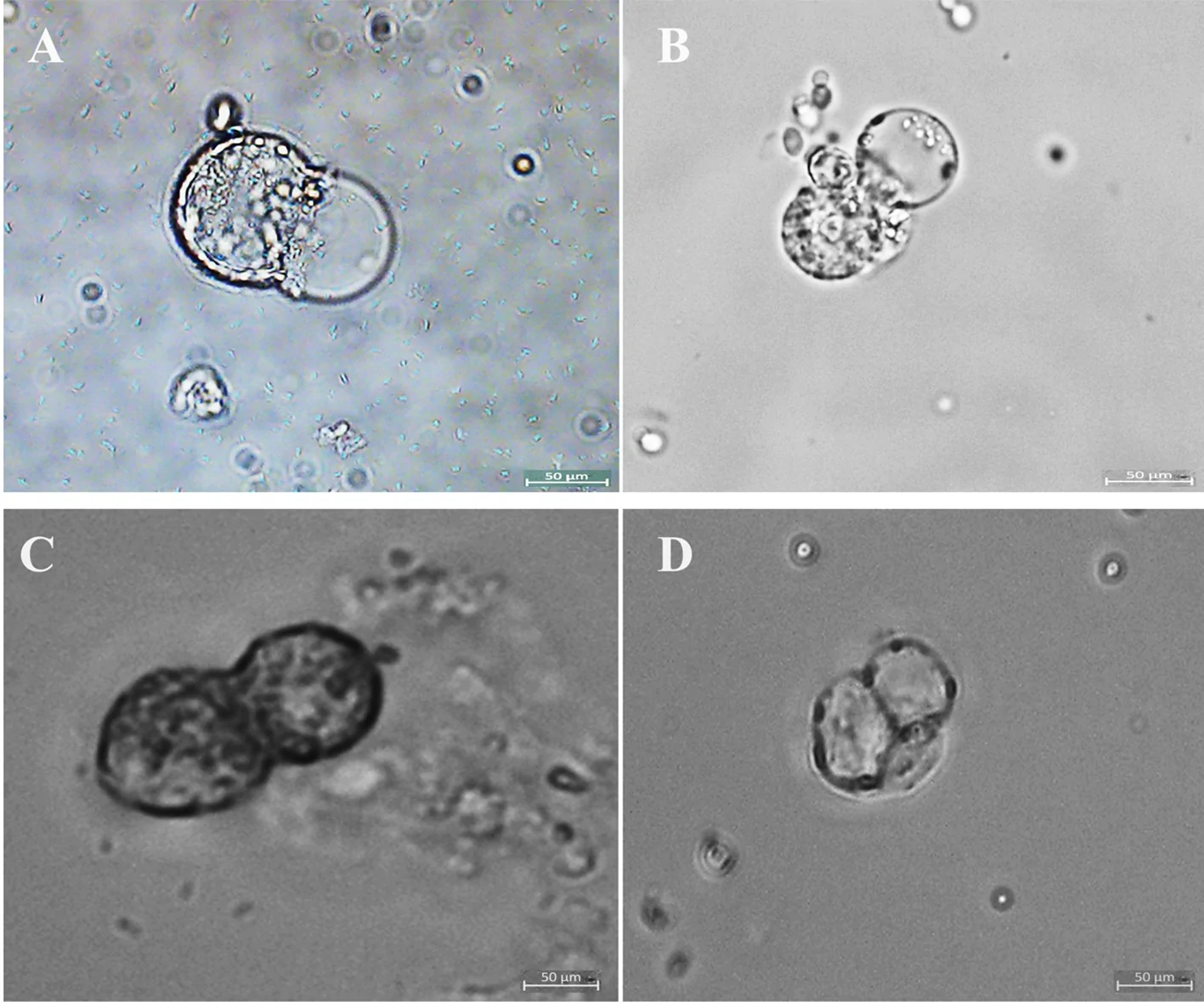
Changes in cell morphology during protoplast culture. **(A)** budding protoplast, **(B)** single cell division, **(C)** first cell division, and **(D)** second cell division. Bar = 50 μm.

The protoplasts, derived from oil palm callus tissue, were cultured in BH3 liquid medium. After 7 days of cultivation, microcolony formation was observed, and after 22 days, larger cell clusters were visible. This lays the groundwork for further development into micro callus tissue (**Figure 3**).

**ABFigure 3:**
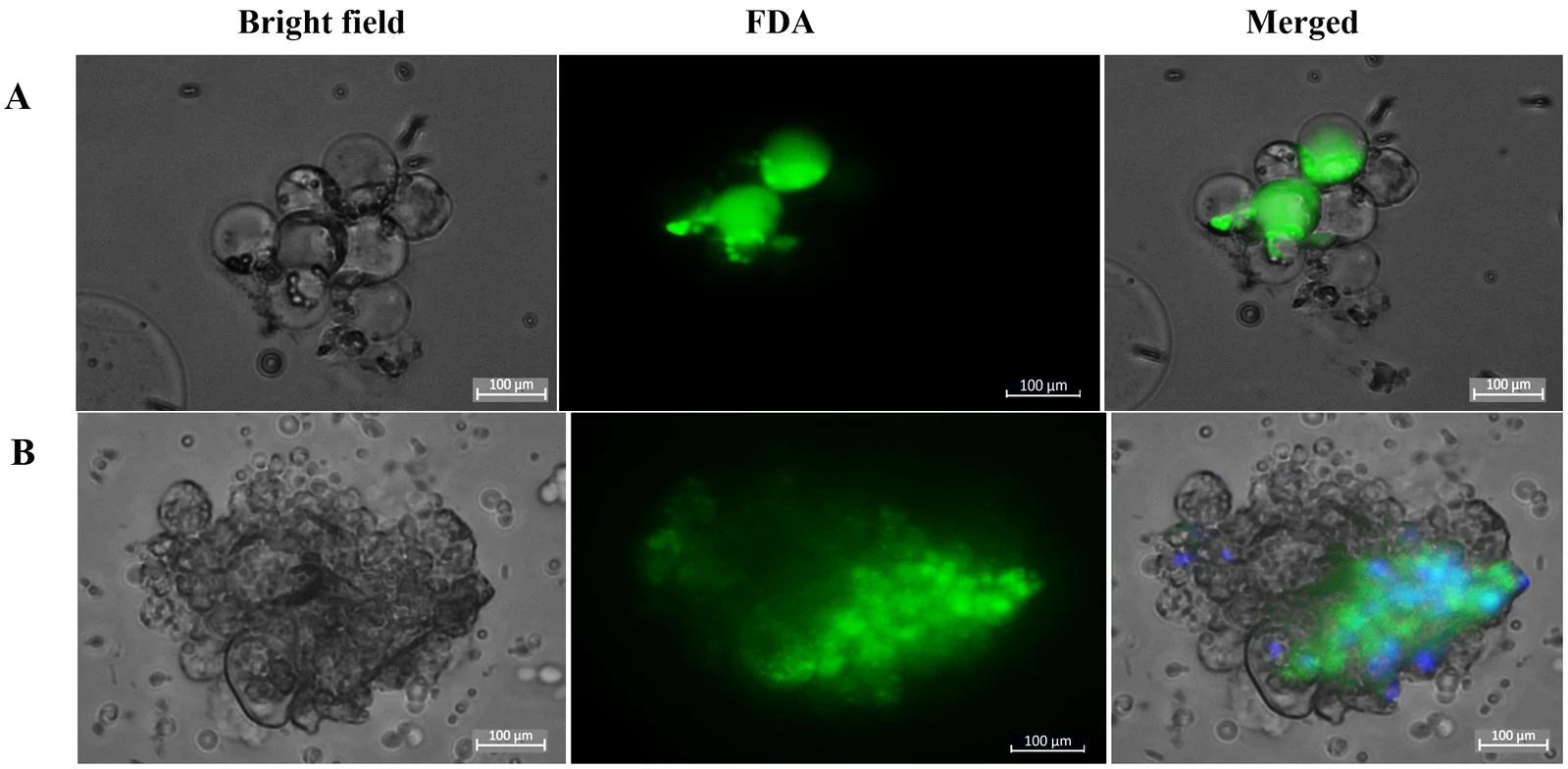
Microcolony formation during protoplast culture in solidified medium supplemented with PGR combination. **(A)** after 7 days of cultivation, **(B)** after 22 days of cultivation. Bar = 100 μm.

### 3.3 Effect of basal media on protoplast development

The data in **Table 1** reveal pronounced differences among culture media in supporting protoplast viability, cell wall regeneration, and subsequent cell cycle progression, indicating strong medium-dependent regulation of early microcolony development.

**Table 1:** Protoplast cell division frequency in different culture media.

Culture medium	Days for cell wall formation	Days for first division	Days for second division	Cell mass (3–6 protoplasts) (%)	Cell mass (>8 protoplasts) (%)	Cell division (%)		Microcolony formation frequency (%)
						First cell division after 5-days culture	Small cell mass after 10-days culture	
BH3 liquid	2.00 ± 0.75	5.00 ± 1.30	8.00 ± 1.70	10.00 ± 1.50	8.00 ± 1.92	76.6 ± 6.0	68.0 ± 5.2	52.3 ± 4.1
Y3 liquid	5.00 ± 1.50	21.00 ± 1.00	28.00 ± 1.50	6.00 ± 2.50	6.00 ± 0.15	16.6 ± 2.0	10.3 ± 1.5	5.6 ± 0.8
MS liquid	7.00 ± 1.00	14.00 ± 1.50	–	8.00 ± 1.50	–	21.0 ± 1.0	14.5 ± 1.8	–
KM liquid	2.00 ± 0.50	9.00 ± 1.00	12.00 ± 1.00	–	–	11.0 ± 1.5	–	–
WPM liquid	6.00 ± 1.50	12.00 ± 1.00	14.00 ± 1.0	–	–	10.0 ± 1.0	3.8 ± 1.5	–
N6 solid	9.00 ± 0.50	21.00 ± 1.00	–	–	–	20.0 ± 1.0	8.5 ± 1.0	–
B5 solid	8 ± 2.00	11.00 ± 1.50	14.00 ± 1.0	–	–	10.0 ± 1.5	2.4 ± 1.0	–
KPR solid	2.00 ± 1.00	3.00 ± 0.30	6.00 ± 1.40	10.00 ± 1.50	10.00 ± 0.50	85.0 ± 5.0	79.0 ± 6.5	66.0 ± 5.8

Values are expressed as mean ± SD (n = 3). “–” indicates no observable response.

Among the tested basal media, KPR provided the strongest overall support for sustained protoplast development, particularly with respect to cell division and microcolony establishment. However, BH3 showed a comparable advantage during the earliest recovery phase, with rapid cell wall regeneration and relatively high early division activity. Therefore, the two media exhibited complementary rather than mutually exclusive advantages during protoplast development. Cell wall regeneration occurred within 2.00 days, followed by the first division (3.00 days) and second division at 6.00 days, resulting in the highest division frequency (0.850) and the formation of robust microcolonies (up to >8 cells). This demonstrates that KPR provides an optimal physiological and nutritional environment for rapid protoplast re-entry into the cell cycle.

In contrast, BH3 liquid medium was particularly effective during the early recovery phase. Cell wall regeneration occurred within approximately 2 days, and the medium maintained a relatively high frequency of subsequent cell division. These findings suggest that BH3 provides favorable conditions for the initial physiological recovery of isolated protoplasts. However, its performance in later developmental stages was lower than that of KPR, particularly for sustained microcolony establishment.

Media such as Y3, MS, KM, and N6 showed delayed or incomplete developmental responses, with notably long lag phases for first division (up to 21 days in Y3 and N6) and absence of later-stage microcolony formation in several cases. In particular, KM and N6 failed to support measurable microcolony expansion, indicating insufficient metabolic or hormonal support for sustained proliferation. Overall, these results demonstrate that rapid protoplast reprogramming and efficient microcolony formation were strongly medium-dependent, with these media exhibiting limited or incomplete developmental competence under the tested conditions.

Microscopic observations further revealed that protoplasts cultured in BH3 medium maintained cellular integrity and sustained active division, ultimately progressing into multicellular aggregates. In contrast, protoplasts maintained in other media frequently lost viability after the initial culture period and failed to sustain successive cell divisions. The enhanced performance observed in BH3 medium may be attributed to its balanced osmotic conditions and organic supplements, including malt extract, which likely supported metabolic reactivation, cell wall reconstruction, and sustained mitotic competence following protoplast isolation.

The efficiency of protoplast culture was strongly influenced by both the basal medium composition and the culture configuration. Specifically, BH3 liquid medium was optimal for early recovery and mitotic activation, whereas KPR nurse culture was highly efficient for microcolony development. Considerable variation in plating efficiency was observed among the tested media and culture systems, indicating a strong influence of nutritional composition and physical culture environment on early protoplast development.

Collectively, these results demonstrate that both medium composition and culture system exert critical effects on protoplast plating efficiency. The superior performance observed in BH3 liquid culture and KPR nurse culture indicates that optimized nutritional balance combined with conditioned cellular microenvironments is essential for enhancing early-stage protoplast regeneration in oil palm protoplasts.

The results in **Table 1** clearly show marked differences among basal media in their ability to support protoplast division and subsequent microcolony formation, indicating medium-dependent differences in sustaining cellular reprogramming and early developmental progression.

The KPR medium exhibited the highest performance, with 85.0% first cell division after 5 days, followed by 79.0 small cell mass formation after 10 days, and the highest microcolony formation frequency (66.0). This confirms that KPR provides the most favorable biochemical and physiological environment for both rapid mitotic activation and continued cellular proliferation. Similarly, BH3 medium showed relatively strong regenerative capacity, recording 76.6% first division, with moderate progression to small cell masses (68.0%) and microcolony formation (52.3%). Although slightly less efficient than KPR, BH3 still supports stable protoplast development.

In contrast, Y3, MS, KM, and N6 media demonstrated markedly reduced efficiency, with first division rates ranging from 11.0% to 21.0%, and a strong decline in later developmental stages. Notably, KM and N6 failed to support detectable microcolony formation, while MS showed only minimal progression and Y3 remained consistently low across all parameters. Overall, the findings indicate that successful protoplast regeneration is highly dependent on basal medium composition, with KPR emerging as the most effective medium for sustaining both early division and microcolony development, while BH3 represents a secondary supportive system. Other media appear suboptimal for maintaining prolonged developmental competence under the tested conditions (**Figure 4**).

**Figure 4:**
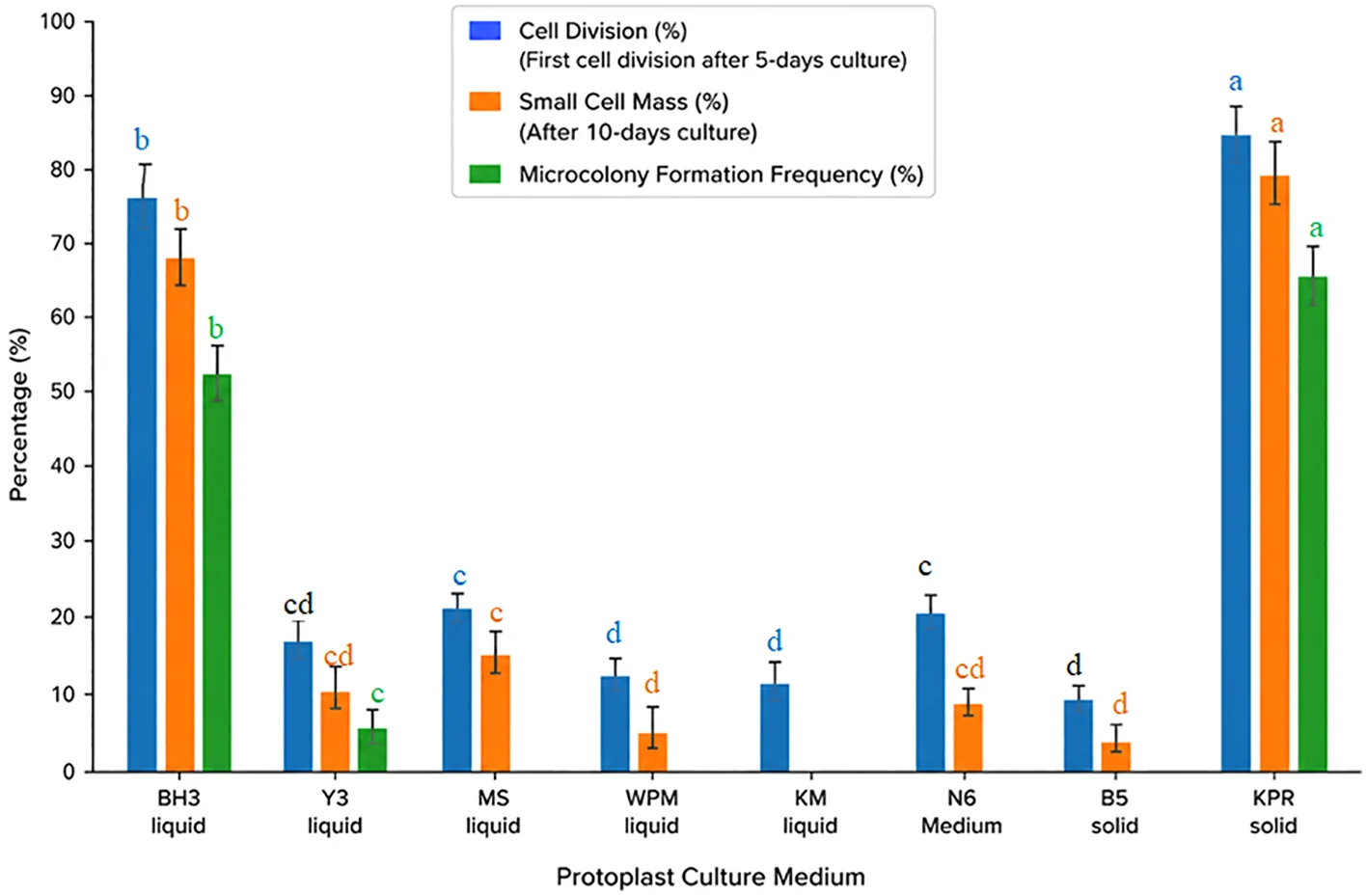
Effect of basal culture media on cell division, small cell mass formation, and microcolony development. (Different lowercase letters above bars within the same parameter indicate significant differences among culture media according to Tukey’s HSD test at P ≤ 0.05).

The comparative results indicate that no single basal medium was uniformly superior across all stages of protoplast development. Instead, culture-medium performance depended strongly on the developmental stage and culture configuration. BH3 liquid medium was particularly effective during the early recovery phase, supporting rapid cell wall regeneration and maintaining relatively high early cell division activity. In contrast, KPR medium showed the strongest overall performance for sustained developmental progression, particularly in terms of first cell division, subsequent cell mass formation, and microcolony establishment. Importantly, the highest plating efficiency was obtained when KPR was combined with the nurse culture system, indicating that the beneficial effect of KPR was enhanced by a supportive cellular microenvironment. Thus, BH3 should be considered the preferred medium for early protoplast recovery and cell wall regeneration, whereas KPR, particularly in combination with nurse culture, was the most effective condition for sustained proliferation and microcolony establishment. These findings indicate that medium selection should be considered stage-specific rather than based on the assumption that one formulation is optimal throughout the entire regeneration process. BH3 is better for early recovery, while KPR is better for sustained developmental progression.

### 3.4 Effect of culture systems on plating efficiency

Basal media significantly affected developmental progression following protoplast isolation. To identify the most suitable culture medium for early protoplast development, oil palm protoplasts were cultured in several basal media, including BH3, Y3, MS, WPM, KM, N6, B5 and KPR. Culture performance was quantitatively evaluated based on the frequency of first cell division and the subsequent formation of small multicellular masses.

The results in **Table 2** clearly demonstrate a strong interaction between basal media composition and culture system in determining protoplast plating efficiency, with highly genotype–medium dependent regeneration behavior.

**Table 2:** Effect of basal medium and culture system on the protoplast plating efficiency.

Basal medium	Liquid culture	Droplet culture	Agar pool culture	Well plate culture	Feeder layer culture	Nurse culture
BH3	1.39 ± 0.14 b	0.00 ± 0.00 k	0.00 ± 0.00 k	0.00 ± 0.00 k	0.00 ± 0.00 k	1.03 ± 0.14 d
Y3	0.13 ± 0.13 ij	0.00 ± 0.00 k	0.00 ± 0.00 k	0.85 ± 0.14 e	0.00 ± 0.00 k	0.00 ± 0.00 k
MS	0.00 ± 0.00 k	0.00 ± 0.00 k	0.00 ± 0.00 k	0.00 ± 0.00 k	1.13 ± 0.17 c	0.00 ± 0.00 k
KM	0.00 ± 0.00 k	0.00 ± 0.00 k	0.00 ± 0.00 k	0.00 ± 0.00 k	0.00 ± 0.00 k	0.00 ± 0.00 k
WPM	0.07 ± 0.11 jk	0.00 ± 0.00 k	0.00 ± 0.00 k	0.00 ± 0.00 k	0.00 ± 0.00 k	0.00 ± 0.00 k
N6	0.00 ± 0.00 k	0.00 ± 0.00 k	0.00 ± 0.00 k	0.00 ± 0.00 k	0.35 ± 0.12 g	0.00 ± 0.00 k
B5	0.00 ± 0.00 k	0.00 ± 0.00 k	0.00 ± 0.00 k	0.27 ± 0.13 gh	0.55 ± 0.13 f	0.00 ± 0.00 k
KPR	0.00 ± 0.00 k	0.00 ± 0.00 k	0.00 ± 0.00 k	0.00 ± 0.00 k	0.21 ± 0.11 hi	3.00 ± 0.15 a

Values are means ± SD (n = 3). Data were analyzed using two-way factorial ANOVA with basal medium and culture system as fixed factors. The basal medium × culture system interaction was significant (P < 0.001). Means followed by different lowercase letters differ significantly according to Tukey’s HSD test at P < 0.05.

Among all tested combinations, BH3 liquid medium exhibited the highest plating efficiency under liquid culture conditions (1.39 ± 0.14), followed by BH3 nurse culture (1.03 ± 0.14). These results demonstrate the superior capacity of BH3-based culture conditions to support early protoplast competence, cell wall regeneration, and mitotic reactivation, suggesting that its nutritional composition effectively sustained cellular activity following enzymatic isolation.

The plating efficiency varied markedly among the tested combinations of basal media and culture systems (**Table 2**). Several media showed limited or no detectable plating efficiency across most culture configurations. In particular, MS and KM showed no measurable response in liquid, droplet, agar pool, or well-plate cultures, while their responses were restricted to feeder-layer culture in MS (1.13 ± 0.17) and remained undetectable for KM. Similarly, N6 showed a limited response in feeder-layer culture (0.35 ± 0.12), whereas B5 produced low to moderate responses in agar pool (0.27 ± 0.13) and feeder-layer cultures (0.55 ± 0.13). Y3 showed a low response in liquid culture (0.13 ± 0.13) and a higher response in well-plate culture (0.85 ± 0.14), while WPM exhibited only a low response in liquid culture (0.07 ± 0.11).

In contrast, substantially higher plating efficiencies were observed with BH3 and KPR under specific culture configurations. BH3 showed the highest response among the liquid-culture combinations (1.39 ± 0.14), followed by nurse culture (1.03 ± 0.14). The most pronounced response overall was obtained with KPR in nurse culture, reaching 3.00 ± 0.15, which was significantly higher than the other treatment combinations. These results further support the stage- and system-dependent interpretation of medium performance. Although BH3 was highly effective in supporting early protoplast recovery under liquid culture, KPR exhibited its greatest benefit when combined with nurse culture. Under this condition, KPR achieved the highest plating efficiency among the tested medium–culture-system combinations. Therefore, the results do not indicate that BH3 and KPR compete as universally superior media; rather, they indicate that their effectiveness depends on the developmental stage and the physical and biological environment in which the protoplasts are cultured.

Culture method significantly influenced division efficiency and microcolony establishment. The results in **Table 3** demonstrate a clear and strong influence of the culture method on protoplast division efficiency and subsequent microcolony development, highlighting the importance of supportive cellular environments for successful early-stage cellular development.

**Table 3:** Effect of culture systems on cultured protoplasts.

Protoplast culture method	Cell division (%)		Microcolony formation frequency (%)
	Division frequency	Small cell mass after 10-days culture	
Liquid culture	23.6 ± 2.4	18.5 ± 2.0	11.0 ± 1.2
Feeder layers culture	3.5 ± 0.6	2.0 ± 0.5	ND
Nurse culture	67.6 ± 5.8	58.0 ± 5.0	44.0 ± 4.5

Overall, the nurse culture system showed the highest efficiency, with 67.6% cell division, followed by 58.0 formations of small cell masses, and a high microcolony formation frequency of 44.0%. This indicates that the presence of nurse cells significantly enhances protoplast viability and mitotic reactivation, likely through the release of growth factors and improved metabolic support.

In comparison, liquid culture exhibited moderate performance, with 23.6% division frequency, but a marked reduction in later developmental stages (18.5% small cell mass and 11.0% microcolony formation). This suggests that while liquid medium can support initial division, it is less effective in sustaining continued proliferation and organized cell aggregation.

In contrast, the feeder layer culture system was largely ineffective, showing only 3.5% cell division, minimal small cell mass formation (2.0%), and no detectable microcolony formation (ND). This reflects poor compatibility under the tested conditions, possibly due to insufficient interaction or suboptimal signaling between feeder cells and protoplasts.

The data clearly indicate that culture method is a critical determinant of regeneration success, with the nurse culture system providing the most conducive environment for sustained division and microcolony formation, while liquid culture offers limited support and feeder layers are largely ineffective in this experimental setup.

### 3.5 Microcallus induction and morphological development

Microcolony formation obtained by protoplast proliferation of oil palm (Microcallus appeared as clustered masses), in BH3 liquid media, after 22–29 days. Visible microcallus developed after prolonged culture under optimized conditions, generally appearing after approximately 8 weeks.

Following prolonged cultivation, visible microcallus structures were induced after approximately eight weeks under optimized conditions (**Figure 5**). However, although microcallus formation was achieved, subsequent callus proliferation remained limited, and regeneration efficiency remained relatively low. This observation indicates that while substantial progress was achieved during early-stage callus development, additional optimization remains necessary to overcome later developmental bottlenecks.

**Figure 5:**
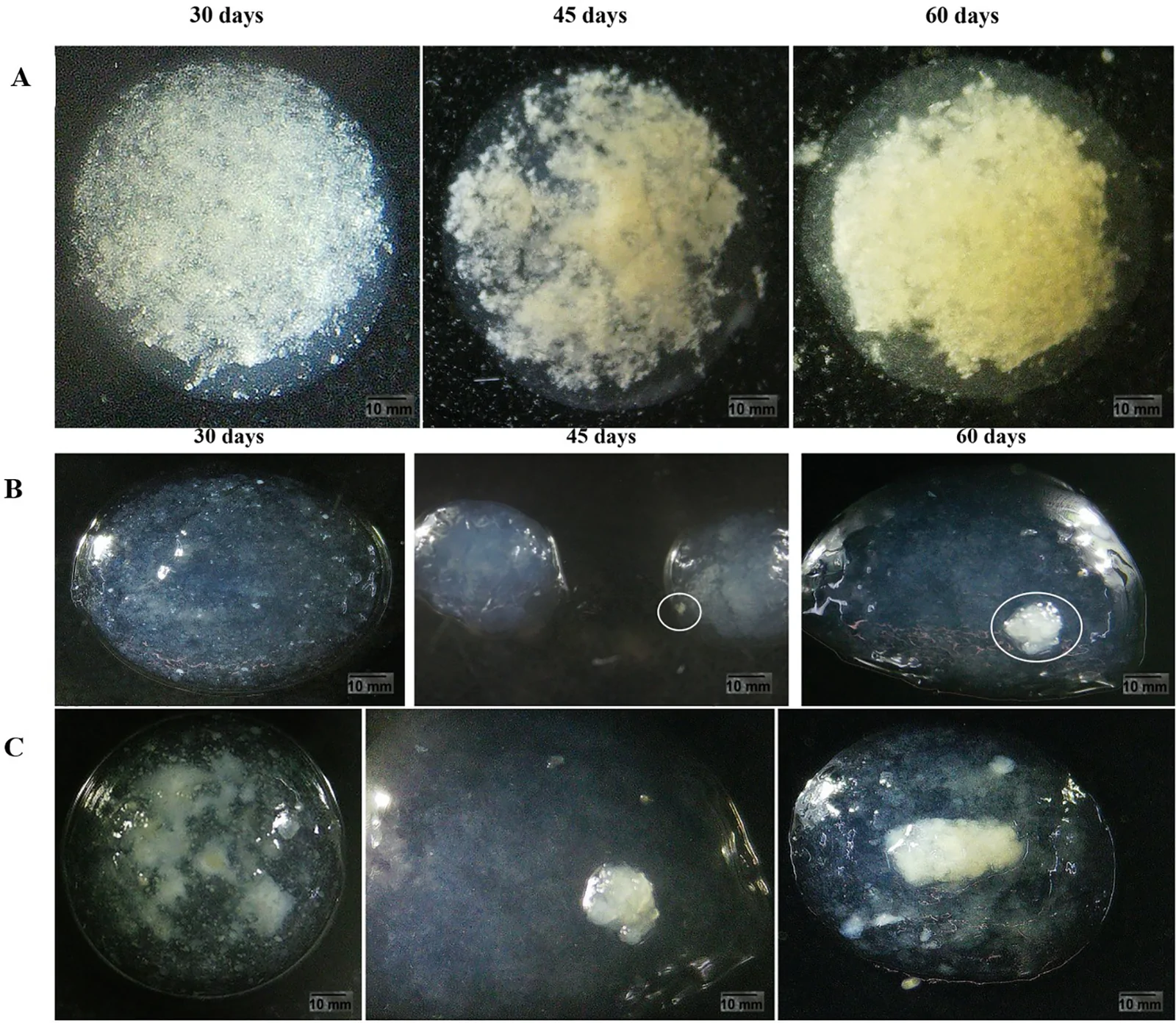
Morphological progression of microcallus formation during protoplast culture. **(A)** protoplasts were cultured in nurse culture system and Y3 basal medium. **(B)** cell colonies were cultured in KPR solid medium, **(C)** early microcallus formation after 60 days.

**Figures 6A, B** illustrates the progressive development of protoplast-derived callus under the optimized culture conditions. In **Figure 6A**, numerous fine and compact microcallus aggregates of different sizes were observed, indicating active cell division and early colony establishment. In **Figure 6B**, continued proliferation led to larger, more organized callus structures, including compact embryogenic regions and actively growing callus clusters. The transition from small microcalli to proliferating embryogenic callus demonstrates successful callus initiation and sustained cellular growth, confirming the effectiveness of the optimized culture system for promoting protoplast regeneration.

**Figure 6:**
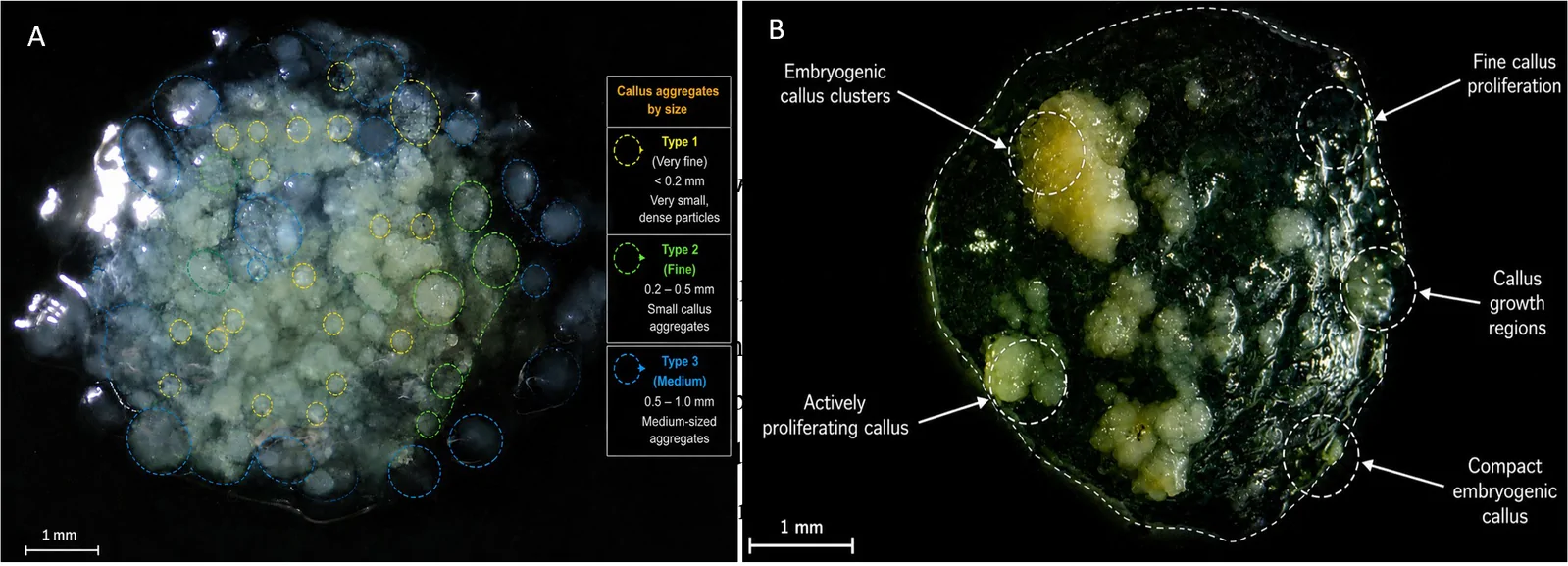
Morphological progression of protoplast-derived callus under optimized culture conditions. **(A)** Formation of fine microcallus aggregates of varying sizes during the early stages of culture under nurse culture. **(B)** Further proliferation and organization of callus tissue, showing actively growing regions and compact embryogenic callus clusters. These observations indicate successful transition from microcolony formation to sustained callus development.

The nurse culture system promoted efficient protoplast proliferation and the formation of fine callus aggregates with variable cluster sizes, ranging from very small compact cell masses to larger developing microcolonies. The observed dense cellular organization and progressive aggregate enlargement indicate active cell division and successful early-stage colony development. The presence of multiple aggregate classes suggests continuous cellular growth and enhanced physiological support provided by the nurse culture environment, which likely improved cell-to-cell signaling and nutrient availability during protoplast development (**Figure 6A**).

The embryogenic callus clusters cultured under *in vitro* conditions exhibited active and heterogeneous proliferation patterns, with measurable differences in aggregate size among the developing callus regions. The presence of compact, actively proliferating callus structures indicates sustained cellular division and successful establishment of embryogenic tissues under the applied culture conditions (**Figure 6B**).

The observed proliferation response may be attributed to the suitability of the nutrient culture medium and the adopted culture system, which provided favorable osmotic and physiological conditions for continuous cell growth and microcallus development. Furthermore, the semi-solid *in vitro* culture environment likely enhanced nutrient availability and cellular organization, promoting progressive expansion of the embryogenic callus aggregates.

### 3.6 Microcallus development and somatic embryogenesis

Based on our oil palm tissue culture prpcedures established priviously (Zhang et al., 2021), microcallus emerged from the KPR solid medium for further proliferation (**Figure 7A**), then it took 90 days to form embyogenic callus, globular embryo and somatic embryos (**Figures 7B–D**); and finaly bud regeneration was observed after another 30 days (**Figures E, F**).

**Figure 7:**
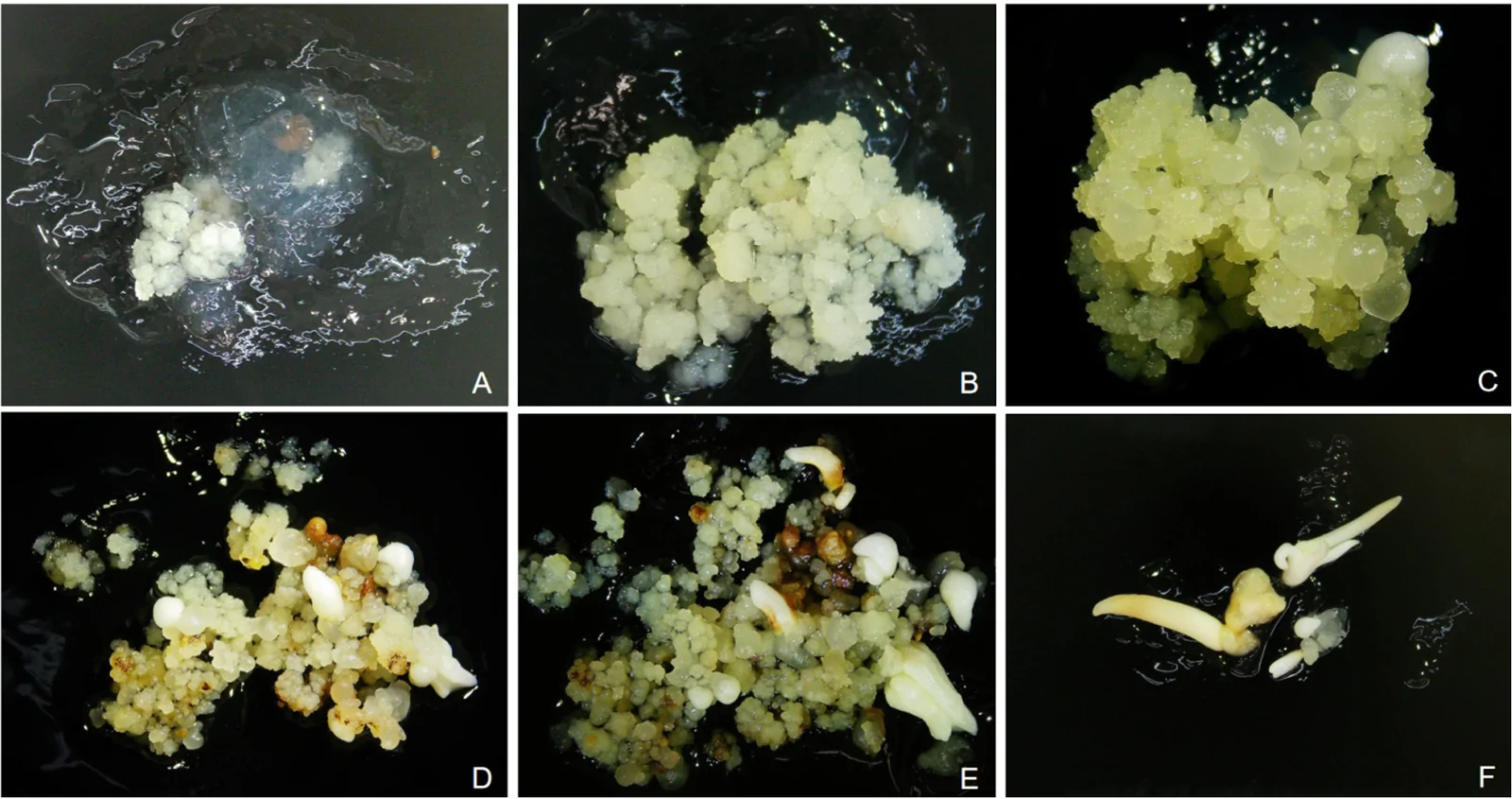
Microcallus development and somatic embryogenesis in oil palm. (**A)** Microcallus proliferation (0 d); **(B)** Embyogenic callus formation (30 d); **(C)** Globular embryo formation (60 d); **(D)** Somatic embryogenesis (90 d); **(E)** Somatic embryo development (105 d); **(F)** Bud regeneration (120 d).

## 4 Discussion

The present study should be considered an early-stage optimization of oil palm protoplast development rather than a complete protoplast-to-plant regeneration or genome-editing platform. Although protoplasts provide an attractive starting material for downstream applications such as genome editing, precision breeding, genetic transformation, and somatic hybridization, none of these applications was experimentally evaluated in the present study. The contribution of this work is therefore to improve the early developmental competence of isolated protoplasts by identifying culture conditions that favor cell-wall regeneration, cell division, plating efficiency, microcolony establishment, and microcallus formation. An efficiently regenerating protoplast population is an important prerequisite for subsequent biotechnology applications because downstream transformation or genome-editing procedures ultimately require the treated cells to remain viable and to progress through cell division and regeneration. However, successful delivery of editing reagents, genetic transformation, somatic fusion, selection of modified cells, and regeneration of edited or hybrid plants involve additional experimental steps that were beyond the scope of the present study. Consequently, the potential application of the optimized conditions to genome editing, precision breeding, or somatic hybridization should be regarded as a future research direction rather than as an experimentally demonstrated outcome of this work.

Efficient protoplast culture and subsequent developmental progression remain major technical challenges limiting the application of advanced biotechnological approaches in tropical palm species. Successful development of isolated protoplasts depends on the coordinated influence of the source tissue, physiological status of the cells, culture medium, osmotic environment, and culture configuration. In the present study, these factors were systematically evaluated to establish a reproducible framework for early-stage development of oil palm (Elaeis guineensis) protoplasts. The results demonstrate that nutrient composition and the physical culture environment substantially influenced protoplast viability, cell wall re-formation, mitotic activation, plating efficiency, and subsequent microcolony and microcallus development, consistent with the recognized importance of these factors in protoplast culture systems (Song et al., 2017; Yang et al., 2023; Liqing et al., 2005; Raikar et al., 2008; Wu et al., 2017).

The initial quality of the protoplast population is an important determinant of subsequent developmental competence. Tissue type, genotype, isolation procedure, and enzyme composition can markedly affect protoplast yield and viability because differences in cell-wall structure and physiological status influence both the efficiency of cell-wall digestion and the ability of released protoplasts to withstand isolation stress (Davey et al., 2005; Raikar et al., 2008; Lord and Gunawardena, 2010). In the present study, leaf tissues from the evaluated accessions consistently provided higher protoplast yields than the other tested tissue sources. This observation is consistent with earlier reports showing that the physiological characteristics of the source tissue strongly influence protoplast isolation efficiency and quality (Ravigadevi et al., 1996). The results therefore emphasize that optimization of the source material should be considered an integral component of any subsequent protoplast culture strategy rather than a separate preliminary step.

Protoplast culture provides an important experimental platform for plant cellular manipulation, regeneration, genetic transformation, and somatic hybridization because the removal of the cell wall allows direct access to individual living cells and their subsequent developmental responses (Davey et al., 2005). However, the successful progression of isolated protoplasts through cell-wall re-formation, cell division, microcolony development, and callus formation is highly dependent on the compatibility between the cellular state and the culture environment. This dependence was particularly evident in the present study through the contrasting responses observed among the evaluated basal media and culture systems.

Among the tested basal media, BH3 and KPR exhibited complementary strengths at different stages of protoplast development. BH3 was particularly effective during the early recovery phase, supporting rapid cell wall regeneration and maintaining relatively high early cell division activity. KPR, in contrast, provided stronger support for continued cell-cycle progression, cell mass formation, and microcolony establishment, particularly when combined with nurse culture. This stage-specific response suggests that medium composition should be matched to the physiological requirements of successive developmental stages rather than treating one medium as universally optimal. This result suggests that BH3 provided a favorable nutritional and osmotic environment for physiological recovery following enzymatic isolation. The relatively complex composition of BH3, including malt extract, may have contributed additional amino acids, vitamins, and organic compounds that supported metabolic recovery and cellular reactivation. Immediately after cell-wall removal, protoplasts are highly susceptible to osmotic and oxidative stress; therefore, an appropriate combination of nutritional support and osmotic regulation is essential for maintaining membrane integrity and allowing re-entry into the cell cycle. Similar importance of medium composition in supporting post-isolation recovery and developmental competence has been reported in other plant systems, including rice, citrus, and rubber (Reed and Argmann, 2021; Li, 2017; Ma, 2021; Sushamakumari et al., 2000).

The superiority of BH3 during the early culture phase also indicates that the most appropriate medium for freshly isolated protoplasts may not necessarily be the optimal medium for all subsequent developmental stages. Rather, the requirements of the cells change as they progress from physiological recovery and cell-wall reconstruction toward active proliferation and multicellular organization. Previous studies have similarly demonstrated that nutritional balance, osmotic regulation, plant growth regulators, and culture conditions must be adjusted according to the developmental stage of cultured protoplasts (Tang et al., 2000). Thus, the present results support a sequential rather than a single-medium approach to oil palm protoplast development.

Culture configuration represented another major determinant of developmental performance. Although BH3 liquid culture provided the most favorable conditions for rapid cell-wall re-formation, osmotic stability, prolonged cell viability, and early cell division, the response to culture configuration was medium-dependent. In particular, KPR medium showed its highest plating efficiency under nurse culture conditions. This result indicates that the physical and biological microenvironment surrounding isolated protoplasts can strongly modify their developmental response. Nurse culture may provide diffusible signaling molecules, phytohormones, peptides, antioxidants, metabolic intermediates, and other conditioning factors that support isolated cells during the physiologically vulnerable early stages of culture (Hu et al., 2015; Masani et al., 2013; Borgato et al., 2007; Kiełkowska and Adamus, 2012).

The enhanced response under nurse culture is particularly relevant to the subsequent progression of oil palm protoplasts. The presence of supporting cells may create a conditioned microenvironment that partially compensates for the absence of the intercellular signals normally present in intact tissues. Such cell-mediated effects have been reported in other recalcitrant plant systems and may be especially important when isolated protoplasts exhibit limited physiological competence or reduced ability to sustain autonomous growth (Masani et al., 2013; Pati et al., 2005). In the present study, nurse culture provided the most rapid overall progression toward clearly visible microcallus formation among the evaluated culture configurations, demonstrating that the optimal condition for early cell division and the optimal condition for subsequent multicellular development are not necessarily identical.

In contrast, droplet culture and agar pool culture did not support sustained protoplast development under the experimental conditions used in the present study. The limited responses observed in these systems may be associated with differences in nutrient diffusion, physical support, cellular communication, or osmotic stability. These results further emphasize the species- and condition-specific nature of protoplast culture optimization. Culture systems that are effective for herbaceous or less recalcitrant species cannot necessarily be transferred directly to woody tropical monocots, for which the physiological requirements of isolated cells may differ substantially (Christey, 2004; Sushamakumari et al., 2000; Dai et al., 2013).

The temporal sequence of developmental events provided additional evidence that the optimized conditions supported progressive cellular reprogramming rather than merely short-term survival. Following isolation, protoplasts underwent cell-wall re-formation within the early culture period, followed by mitotic activation and progressive formation of multicellular aggregates and microcolonies. Under the most favorable conditions, this progression continued to the formation of visible microcallus structures. The ability of the isolated cells to pass through these developmental stages indicates that the protoplasts retained sufficient physiological and developmental competence after enzymatic isolation to re-enter the cell cycle and establish multicellular growth. Similar developmental transitions have been recognized as critical intermediate stages in oil palm protoplast culture (Masani et al., 2013; Guo et al., 2023).

An important feature of the present study is that rapid progression toward microcallus formation resulted from the coordinated optimization of source-tissue developmental status, basal medium, and culture configuration rather than from a single superior culture condition. The experimental results showed that BH3 liquid medium was particularly effective during the initial recovery phase, promoting rapid cell-wall re-formation, maintaining osmotic stability and cell viability, and supporting early and sustained cell division. Following this stage, transfer to a suitable solid Y3-based medium favored continued multicellular development, whereas KPR medium combined with nurse culture provided the most rapid progression toward clearly visible microcallus formation among the culture systems evaluated. Accordingly, the results support a stage-specific framework in which BH3 liquid culture facilitates protoplast recovery and mitotic activation, followed by solid Y3-based culture for continued development and nurse culture with KPR for accelerated microcallus formation. This integrated sequence provides a practical framework for early oil palm protoplast development under the conditions evaluated, while further optimization remains necessary to sustain microcallus proliferation and achieve subsequent embryogenic development and plant regeneration.

Compared with the pioneering regeneration system of Masani et al. (2013), the present study addresses a different but complementary stage of the oil palm protoplast regeneration pathway. Masani et al. (2013) demonstrated the first successful regeneration of viable oil palm plants from protoplasts isolated from cell suspension cultures and established the importance of medium composition, plant growth regulators, and culture technique. Their system employed agarose bead culture with Y3A-based medium and subsequently achieved plant regeneration through successive developmental stages, with viable plants reported approximately 14–17 months after protoplast isolation (Masani et al., 2013). In contrast, the present study systematically compared multiple basal media and culture configurations while also considering the developmental characteristics of the source tissues, with particular emphasis on accelerating the early stages from protoplast recovery through cell division, microcolony formation, and visible microcallus development. Therefore, the novelty of the present work lies not in replacing the complete regeneration system established by Masani et al. (2013), but in defining an integrated, stage-specific culture framework that facilitates rapid and reproducible progression toward the microcallus stage under the conditions evaluated in the present study.

The quantitative outcomes of the present study should be interpreted in relation to the specific developmental endpoints evaluated rather than as a direct percentage improvement over previously published whole-plant regeneration protocols. The experimental designs and reported endpoints of previous studies, particularly those describing complete regeneration, are not directly equivalent to the early-stage parameters quantified here. Within the present experimental framework, however, clear quantitative differences were observed among culture conditions. KPR medium produced an 85.0 ± 5.0% first-cell-division response and a 66.0 ± 5.8% microcolony formation frequency, compared with 76.6 ± 6.0% and 52.3 ± 4.1%, respectively, in BH3. Furthermore, KPR combined with nurse culture produced the highest plating efficiency observed in the medium × culture-system comparison (3.00 ± 1.34%), while nurse culture as a culture configuration produced 67.6 ± 5.8% cell division and 44.0 ± 4.5% microcolony formation. These quantitative differences demonstrate that the principal advance of the present work is the identification of stage- and system-dependent performance rather than a claim of superiority over the complete regeneration efficiency reported previously. We have therefore avoided calculating a potentially misleading percentage improvement against studies using different experimental designs and endpoints.

An important limitation of oil palm protoplast culture is the prolonged time required for progression from early cell proliferation to stable callus development, embryogenic competence, and ultimately plant establishment. In the present study, visible microcallus structures generally appeared only after approximately eight weeks, and subsequent callus proliferation remained limited, highlighting a major developmental bottleneck beyond the early stages of protoplast recovery. Although optimization of culture media, osmotic conditions, antioxidants, and culture configuration may improve this transition, molecular approaches targeting cellular reprogramming and regeneration competence may provide an additional strategy to accelerate the process. Previous work in oil palm demonstrated that microcalli derived from protoplasts can subsequently undergo somatic embryogenesis and plant regeneration following extensive optimization of culture conditions and successive subculturing steps (Masani et al., 2013). At the molecular level, regeneration-promoting transcription factors such as WUSCHEL (WUS), BABY BOOM (BBM), and LEAFY COTYLEDON (LEC) have been implicated in cellular reprogramming, acquisition of embryogenic competence, and somatic embryogenesis in different plant systems (Horstman et al., 2017; Fambrini et al., 2022; Yuan et al., 2024). Their controlled or inducible expression could therefore be investigated in oil palm to determine whether activation of regeneration-associated developmental programs can shorten the transition from protoplast-derived microcalli to embryogenic tissues and subsequently improve plantlet recovery. However, such molecular interventions were not evaluated in the present study and should be considered a future research direction rather than a demonstrated component of the current protocol. Recent advances further suggest that combining morphogenic regulators with appropriate hormonal and cellular environments may be more effective than manipulating individual regeneration factors alone (Jang et al., 2026). A combination of stage-specific culture optimization with targeted molecular manipulation may ultimately provide a more efficient strategy for overcoming the long regeneration cycle characteristic of oil palm.The relatively limited progression beyond the microcallus stage also highlights an important biological constraint that remains to be addressed. Complete plant regeneration requires additional developmental transitions involving sustained microcallus proliferation, acquisition or maintenance of embryogenic competence, appropriate hormonal regulation, and subsequent somatic embryogenesis. Oxidative stress, cellular senescence, altered hormonal responsiveness, and metabolic imbalance may interfere with these later stages and have been recognized as important constraints in recalcitrant palm regeneration systems. Accordingly, the formation of microcalli in the present study should be regarded as an important intermediate achievement toward regeneration rather than as evidence of complete plant regeneration (Masani et al., 2013; Guo et al., 2023).

The prolonged progression from callus to regenerated plants is also a recognized bottleneck in plant regeneration systems, particularly in recalcitrant crops. Beyond further refinement of culture conditions, molecular manipulation of developmental regulators may provide an additional strategy for improving regeneration competence. Transcription factors such as WUSCHEL (WUS), BABY BOOM (BBM), and LEAFY COTYLEDON (LEC) are recognized as important regulators of cellular reprogramming, somatic embryogenesis, and plant regeneration, and their ectopic expression or targeted activation has enhanced regeneration responses in several plant systems (Horstman et al., 2017; Lowe et al., 2016; Maher et al., 2020). Recent studies have also demonstrated the importance of WUS in protoplast regeneration and identified BBM-related regulatory activity during somatic embryogenesis in oil palm, suggesting that manipulation of such developmental regulators could be explored to improve the transition from microcallus to embryogenic structures and regenerated plantlets. However, these approaches should be regarded as prospective strategies for oil palm rather than established components of the present protocol, and their effectiveness, developmental specificity, and potential effects on regeneration fidelity would require experimental validation.

The scope of the present study was deliberately focused on the early developmental progression of oil palm protoplasts, rather than on complete protoplast-to-plant regeneration. The experimental evaluation covered cell-wall regeneration, cell division, plating efficiency, microcolony formation, and progression toward microcallus formation. Although these stages represent important developmental steps in establishing a competent protoplast culture, the present study did not demonstrate subsequent somatic embryogenesis, shoot formation, or whole-plant regeneration. Accordingly, the term regeneration in the context of the present work refers specifically to early cellular recovery and developmental progression toward microcallus formation and should not be interpreted as complete plant regeneration. Previous studies have demonstrated that oil palm protoplasts can be advanced beyond these stages toward somatic embryogenesis and plant regeneration. Therefore, the contribution of the present study is not to claim the first complete regeneration of oil palm from protoplasts, but to provide a systematic, stage-specific comparison of culture configurations and basal media during the early developmental phase. The optimized conditions identified here may serve as a useful starting point for subsequent studies integrating embryogenic induction, shoot development, and whole-plant regeneration. Further optimization will be required to connect the early developmental stages characterized in this study with successful somatic embryo formation, shoot regeneration, and recovery of fertile plants.

Taken together, the present findings indicate that early oil palm protoplast development is strongly dependent on stage-specific coordination of the culture medium and culture environment. BH3 liquid medium was particularly effective during the initial recovery phase, supporting rapid cell-wall re-formation, osmotic stability, cell viability, and early cell division, whereas subsequent culture on a suitable solid Y3-based medium favored continued developmental progression. Notably, KPR medium combined with nurse culture provided the most rapid progression toward visible microcallus formation under the conditions evaluated, emphasizing the importance of a conditioned cellular environment during later early-developmental stages. Further work should focus on sustaining microcallus proliferation and improving its transition to embryogenic callus and regenerated plantlets through optimization of osmotic conditions, plant growth regulator combinations, antioxidant supplementation, and feeder-cell conditioning. Thus, the present study provides a defined early-stage framework for oil palm protoplast development that can support subsequent efforts toward complete regeneration and future biotechnological applications.

Protoplast regeneration will be very useful in seedling cloning and somatic hybridization; while combined with protoplast gene-editing, it could be a new solution for DNA-free gene-editing and germplasm creation for tropical palm trees. The Malaysian Palm Oil Board (MPOB) already established the DNA-free CRISPR/Cas9 genome editing system for oil palm protoplasts using multiple ribonucleoproteins (RNPs) complexes (Norfaezah et al., 2024). Fortunately, our team also established the protoplast gene-editing system for both coconut (Batool et al., 2025) and betel nut (Nie et al., 2025). We believe we can combine the protoplast gene-editing and regeneration together for breeding for these tropical palm trees, and we are still working on it.

## 5 Conclusion

The present study focused on the early developmental stages of oil palm protoplast culture, and optimized a stage-specific culture strategy for quick microcallus formation; Briefly, BH3 liquid medium was most suitable for early protoplast recovery and cell wall regeneration, whereas KPR solid medium in combination with nurse culture system, was more effective for sustained cell division, microcolony and microcallus formation. Combined with previous oil palm tissue culture system, further somatic embryogenesis and bud regeneration were established successfully. This work not only promoted the protoplast regeneration of oil palm, but also provides a promising strategy for other tropical palm trees such as coconut and betel nut.

## Data Availability

The original contributions presented in the study are included in the article/**Supplementary Material**. Further inquiries can be directed to the corresponding authors.
