# Supplementary material for: Optimization of protoplast cell division and microcallus formation toward somatic embryogenesis in oil palm
Source: Front Plant Sci. 2026 Oct 7;17:1952622. doi: 10.3389/fpls.2026.1952622 (PMC13684913; doi:10.3389/fpls.2026.1952622)
Supplement: Supplementary file 1 [file DataSheet1.docx]

**Supplementary Materials**

**Table S1.** Enzyme and buffer solution**s** for protoplast isolation.

| **Solution Name** | **Solution Composition** | **Volume** | **Storage** | **Function** |
| --- | --- | --- | --- | --- |
| Buffer solution | 130g D-Mannitol, 4.264g MES, 1.491g KCL, 1.4701g CaCl_2_.2H_2_O, 0.246g MgSO4.7H2O, 0.0272g KH_2_PO_4_ (PH 5.8) | 1000 ml | Stored at −20 ◦C and thawed before use | Release of protoplasts |
| Enzyme solution | 0.3% Cellulases Onozuka R10, 0.15% Macerozyme R-10, 0.20% Pectinase, BSA 200µl, AMP 10µl, ß-Mercaptoethanol 7.14µl | 20 ml | Freshly prepared | Digestion of cell wall material |
| W5 | 9.0g NaCl, 18.4g CaCl_2_, 0.37g KCL, 0.9g Glucose, 0.3g MES (PH 5.8) | 1000 ml | 4 °C | Purification, release and washing protoplasts |

**Table S2.** Composition of the media used for protoplast culture.

| **Medium code** | **Basal medium** | **Type of media** | **Composition of media** | **Plant growth regulators (PGR)** |
| --- | --- | --- | --- | --- |
| CW1 | MS | Liquid | 30 g/L glucose, 3.0 g/L glutamine | 2 mg/L 6-BA + 0.2 mg/L NAA |
| CW2 | MS | Liquid | 30 g/L glucose, 1.5 g/L glutamine | 2 mg/L 6-BA + 0.1 mg/L NAA |
| CW3 | MS | Liquid | 45 g/L glucose | 1 mg/L 6-BA + 0.1 mg/L NAA |
| CW4 | MS | Embedded in semi-solid agarose | 1.2 % (w/v) MES + 21 % (w/v) sucrose + 0.4 M glucose + 0.3 % agarose | 100 mg/L 2,4-D + 1.5 mg/L 6-BA |
| CW5 | MS | Embedded in semi-solid phytogel | 30 g/L Sucrose, 0.4 M mannitol | 2 mg/L NAA + 1 mg/L 6-BA |
| CW6 | MS | Embedded in solidified agarose | 1.2 % (w/v) MES + 21 % (w/v) sucrose + 0.4 M glucose + 0.6 % agarose | 100 mg/L 2,4-D + 3 mg/L NAA |
| CW7 | MS | Liquid | 1g/L Casine + 0.4 M mannitol | 2.5 mg/L NAA |
| CW8 | MS | Liquid | 30 g/L sucrose, 175 mg/L KH2PO4 + 100 mg/L inositol + 200 mg/L glutamine + 2 mg/L glycine | 1.5 µM Zn/L + 4.5 µM 2,4-D |
| CW9 | MS (without NH_4_NO_3_) | Liquid | 30 g/L glucose + 3.0 g/L glutamine + 9 % mannitol (0.5 M) | - |
| CW10 | WPM | Liquid | 30 g/L glucose, 3.0 g/L glutamine | 2 mg/L 6-BA + 0.2 mg/L NAA |
| CW11 | WPM | Liquid | 30 g/L glucose | 2 mg/L 6-BA + 0.2 mg/L NAA |
| CW12 | WPM | Liquid | 30 g/L glucose, 1.5 g/L glutamine | 2 mg/L 6-BA + 0.3 mg/L NAA |
| CW13 | WPM | Liquid | 1g/L Casine + 0.4 M mannitol | 2.5 mg/L NAA |
| CW14 | WPM | Embedded in semi-solid agarose | 1.2 % (w/v) MES + 21 % (w/v) sucrose + 0.4 M glucose + 0.3 % agarose | 100 mg/L 2,4-D + 1.5 mg/L 6-BA |
| CW15 | WPM | Embedded in solidified agarose | 1.2 % (w/v) MES + 21 % (w/v) sucrose + 0.4 M glucose + 0.6 % agarose | 100 mg/L 2,4-D + 3 mg/L NAA |
| CW16 | KM | Liquid | 30 g/L glucose + 3.0 g/L glutamine + 9 % mannitol (0.5 M) | 10 µm NAA + 10 µm 2,4-D + 10 µm 6-BA + 10 µm ZT + 2 µm IBA |
| CW17 | KM | Solidified | 0.06 M sucrose + 2.3 % Gelrite | 3.1 µm 6-BA + 4.53 µm 2,4-D + 0.91 µm ZT |
| CW18 | KM | Embedded in solidified agarose | 10 % glucose + 1.2 % agarose | 0.5 mg/L 2,4-D |
| CW19 | Y3Y3 | Liquid | 1.2 % (w/v) MES + 10 mL of 21% (w/v) osmoticum solution (sucrose, glucose or mannitol + 0.4 M glucose + 0.6 % agarose | 100 mg/L 2,4-D + 3 mg/L NAA |
| CW20 | Y3 | Embedded in solidified agarose | 1.2 % (w/v) MES + 10 mL of 21% (w/v) osmoticum solution (sucrose, glucose or mannitol + 0.4 M glucose + 0.6 % agarose | 100 mg/L 2,4-D + 3 mg/L NAA |
| CW21 | Y3^*^ | Liquid | - | 2 mL 2,4-D |
| CW22 | Y3^*^ | Embedded in semi-solid agarose | - | 2 mL 2,4-D |
| CW23 | BH3 | Liquid | MT basic culture medium + 0.15 mol/L sucrose + 500 mg/L malt extract | - |
| CW24 | N6^#^ | Agarose bead | 30 g/L Sucrose, 500 mg/L Casein, 100 mg/L Myo-inositol | 2 mL 2,4-D |
| CW25 | B5^#^ | Agarose bead | 20 g/L Sucrose, 500 mg/L Casein, 500 mg/L Glutamine | 1.5 mL 2,4-D + 2 mL BAP + 0.5 mg Kinetin |
| CW26 | MSA | Phytagel | 30 g/L Sucrose, Alginine, Sorbito, Glycine | 1.5 mL 2,4-D + 2 ml BAP |
| CW27 | N6A | Phytagel | 30 g/L Sucrose, Alginine, Sorbitol, Glycine | 1.5 mL 2,4-D + 2 ml BAP |
| CW28 | Y3A | Phytagel | 30 g/L Sucrose, Alginine, Sorbitol, Glycine | 1.5 mL 2,4-D + 2 ml BAP |
| CW29 | KPR^*^ | Liquid | - | 2 mL 2,4-D |
| CW30 | KPR^*^ | Embedded in semi-solid agarose | - | 2 mL 2,4-D |

KM- according to [38] Kao and Michayluk, 1975; B5 according to [39] Gamborg et al., 1968; KPR- according to [40] Abdullah et al., 1986; # - Agarose bead; * - feeder layers and nurse cultures

**Table S3**. Combination of plant growth regulators used in microcallus formation.

| Medium code | Medium type | Medium type | Plant growth regulators (PGR) |
| --- | --- | --- | --- |
| MC1 | Solidified | MS | 0.2 mg/L TDZ + 2.0 mg/L NAA |
| MC2 | Solidified | MS | 2.0 mg/L NAA + 1.0 mg/L 6- BA |
| MC3 | Solidified | MS | 1.0 kinetin + 0.5 mg/L 2,4-D |
| MC4 | Solidified | Y3 | 0.2 mg/L TDZ + 2.0 mg/L NAA |
| MC5 | Solidified | Y3 | 2.0 mg/L NAA + 1.0 mg/L 6- BA |
| MC6 | Solidified | Y3 | 1.0 kinetin + 0.5 mg/L 2,4-D |
| MC7 | Solidified | N6 | 0.2 mg/L TDZ + 2.0 mg/L NAA |

**Figure S1.** Comparison of different protoplast culture systems: (a) liquid culture; (b) droplet culture; (c) agar pool culture, (d) well-plate culture, (e) feeder layer system, and (f) nurse culture.

**c**

**b**

**f**

**e**

**d**

**Figure S2.** Formation of culture medium and addition of protoplasts. (A) well culture plate, (B) agar pool. a Before addition of protoplasts. b After addition of protoplasts (C) Newly isolated protoplasts on semi-solidified medium (nurse culture) containing nurse cells of suspension cells of callus of oil palm, (D) Newly isolated protoplasts on a membrane overlaying semi-solidified medium (feeder layers culture) containing nurse cells of suspension cells of callus of oil palm.
